# Racial Differences of Pediatric Hypertension in Relation to Birth Weight and Body Size in the United States

**DOI:** 10.1371/journal.pone.0132606

**Published:** 2015-07-15

**Authors:** Liwei Chen, Neal Simonsen, Li Liu

**Affiliations:** 1 Department of Public Health Sciences, Clemson University, Clemson, South Carolina, United States of America; 2 Consultant at School of Public Health, Louisiana State University Health Sciences Center, New Orleans, Louisiana, United States of America; 3 Department of Population, Family, and Reproductive Health, Johns Hopkins Bloomberg School of Public Health, Baltimore, Maryland, United States of America; 4 Department of International Health, Johns Hopkins Bloomberg School of Public Health, Baltimore, Maryland, United States of America; Weill Cornell Medical College, QATAR

## Abstract

**Background:**

The prevalence of hypertension is known to differ by racial group in adults in the United States (US), but findings in children are scarce and inconsistent. The objective of this study was to assess the racial differences in pediatric hypertension and to explore whether these differences, if any, can be explained by low birth weight (LBW) and obesity.

**Methods:**

Analyses were performed for participants aged 8–17 years (N = 9,250) included in the 1999–2010 National Health and Nutrition Examination Survey. Multivariate logistic regressions and weighted analysis were carried out considering the complex survey design.

**Results:**

Compared to non-Hispanic White youth, the crude prevalence of hypertension was significantly higher in non-Hispanic Blacks (7.1% vs. 5.6%; P = 0.04), but not in Mexican Americans (5.4% vs. 5.6%; P = 0.77). Blacks also had higher rates of LBW (14.6% vs. 5.9%; P <0.001) and obesity (22.9% vs. 15.8%; P <0.001) than Whites. In stratified analysis by age-sex groups, the Black-White difference in hypertension prevalence was only significant in boys aged 13–17 (9.6% vs. 6.6%). After controlling for age, Black boys had a 51% higher odds of having hypertension (Odds ratio = 1.51; 95% confidence interval: 1.03, 3.43; P = 0.04) compared to White youth at ages 13–17. This racial difference persisted with additional adjustment for birth weight (odds ratio (OR) = 2.00; P = 0.02) and for current body mass index (OR = 1.50; P = 0.04). Mexican American youth had no difference in hypertension prevalence as compared to White youth after adjusting for age, sex, birth weight and obesity (Odds ratio = 0.82; P = 0.16) and in age-sex stratified subgroups.

**Conclusions:**

Non-Hispanic Black adolescent boys have a significantly higher hypertension rate than their non-Hispanic White counterparts in the US. This racial difference cannot be explained by LBW and current obesity status within the Black population.

## Introduction

It is well documented that the prevalence of adult hypertension is different by race in the US with Non-Hispanic Blacks having a higher prevalence compared to Non-Hispanic Whites [1]. Considering the tracking of blood pressure (BP) levels from childhood to adulthood [2, 3], it is critical to examine racial disparities among children and adolescents and to better understand the associated risk factors in order to narrow the racial gaps among adults.

Relatively few studies have investigated racial differences in pediatric hypertension (PHT), with mixed findings [4–8]. Most of the earlierstudies characterized racial differences in hypertension among children and adolescents as non-significant [4, 6–8]. However, Rosner and colleagues reported a significant racial difference between Hispanic and White boys, which persisted after controlling for body mass index (BMI) [5]. Body size and sex appeared to modify the association between race and PHT [5]. Significant differences were also observed in subsequent studies by Lo et al. [9] and Rosner et al. [10] for Black and by Shay et al. [11] for both Black and Hispanic youth compared to their White counterparts. In addition, low birth weight (LBW) has been linked with high risk of adult hypertension [12, 13], although the evidence was less consistent among children and adolescents [14, 15] The role of LBW in racial disparities in PHT is unclear.

The objectives of this study were to identify racial differences in the prevalence of PHT in the US and to explore whether such differences can be explained by body size and birth weight using the most updated nationally representative data from the National Health and Nutrition Examination Survey (NHANES) 1999–2010.

## Methods

### Study Population

The study population included children and adolescents aged 8–17 years who participated in the NHANES 1999–2010. Details of the NHANES design and procedures are available on the Center for Disease Control and Prevention (CDC) website [16]. In brief, the 1999–2010 NHANES included 6 cross-sectional surveys of the non-institutionalized US population. These surveys applied a stratified multistage probability sampling design. Low-income populations, adolescents, seniors, Non-Hispanic Blacks, and Hispanics were over-sampled. Each survey consisted of a household interview and a physical examination in a fully equipped mobile examination center. The procedures and protocols of NHANES 1999–2010 were approved by the institutional review board of the CDC/ National Center for Health Statistics (NCHS). Informed consent was obtained from study participants or their guardians where appropriate [16]. This study was a secondary analysis using the de-identified data released from the CDC, therefore, no additional consent procedures were carried out. For the current study, individuals who self-identified as “Other Hispanics” (6.37%) or “Other Race” (6.73%) were excluded due to small sample size. All the data are available at the CDC NHANES website and are open to public access **(** http://wwwn.cdc.gov/nchs/nhanes/search/nhanes11_12.aspx **).**

### Measurement of BP and Definition of PHT

For children and adolescents aged 8 years or above, BP was measured in a seated position following 5 minutes of quiet rest during the physical examination. For each individual, up to three completed BP measurements were taken at one visit by a physician using a mercury sphygmomanometer with an appropriate size cuff. The measuring procedure followed the American Heart Association standard protocol [17]. Quality control of BP measurements was reinforced in the NHANES [18]. The mean of the three measurements were used to determine PHT status.

PHT was defined as a systolic blood pressure (SBP) or diastolic blood pressure (DBP) ≥ 95th percentile of age-, sex-, and height-specific reference level for the US population, according to the Fourth Task Force Report [19]. A height z-score relative to the 2000 (most recent) CDC growth charts was calculated for each child according to the CDC Box-Cox transformation estimation procedure [20]. The age-, sex-, and height-specific BP percentile was then calculated using the methods provided by the Fourth Task Force Report [19], applying the following steps: 1) compute the expected BP value for a given age, sex, and height z score; 2) convert the participant’s observed BP into a Z score; and 3) convert the BP Z score into a percentile.

Individuals who were taking antihypertensive medications were also considered as having PHT. Antihypertensive medication use was determined by utilizing information from the Prescription Medications questionnaire of NHANES, which was verified by survey interviewers who asked to see the medication containers when possible. It was defined as reported use of at least one of the following classes of medications: angiotensin converting enzyme inhibitor, beta-adrenergic blocking agents, calcium channel blocking agents, diuretics, angiotensin II inhibitors, other antihypertensive agents (other antiadrenergic agents, vasodilators, renin inhibitors, agents for hypertensive emergencies, agents for pulmonary hypertension, and antihypertensive combinations). Antihypertensive medications were classified using the Multum Lexicon Drug Database **(** http://www.multum.com/Lexicon.htm **)**.

### Measurements of Race, Age, Body Size, and Birth Weight

Race was self-reported during the household interview. It was derived by combining responses to questions on race and Hispanic origin and classified into the following categories: Non-Hispanic Whites, Non-Hispanic Blacks, Mexican Americans, Other Hispanics, and Other Race. Age was grouped into two categories: children aged 8–12 years and adolescents aged 13–17 years.

Anthropometric measurements were conducted by a trained examiner following the standard protocol in the mobile examination center during the physical examination [21]. Body weight was measured to the nearest 0.05 kg using the Toledo digital scale while wearing underwear, a disposable gown, and foam slippers. Standing height was measured to the nearest 0.1 cm by using a fixed stadiometer with a vertical backboard and a moveable headboard. BMI was calculated as weight (kilogram) divided by height (squared meters) and classified into three categories: normal weight (<85.0^th^ percentile of sex-and-age-specific BMI), overweight (85.0^th^ -94.9^th^ percentile of sex-and-age-specific BMI) and obesity (≥95.0^th^ percentile of sex-and-age-specific BMI).

Birth weight (in pounds and ounces) was self-reported and collected during the interview from participants aged 15 years or younger and their guardians. Previous studies have shown that maternal- and self-reports of birth weight and LBW status have high validity [22, 23]. The specificity of self-reported birth weight below 3,000 grams was 93% among middle-aged and elderly women participating in the Danish Nurse Cohort Study [23]. The specificity is likely to be higher among children and adolescents given a shorter recall period [24]. Birth weight at or below 5.5 pounds or approximately 2,500 grams was defined as LBW.

### Statistical Analyses

Results of descriptive analysis were expressed as mean or percent, with the corresponding standard error (SE) presented. Odds ratio (OR), 95% confidence interval (CI) and *P* values based on multivariable logistic regressions were conducted to evaluate the independent association between race and PHT after controlling for BMI, LBW and their interactions. This was realized through a stepwise modeling approach, in which race, BMI categories, LBW, and the interaction between BMI categories and birth weight categories were added to the regressions sequentially. Age categories were also adjusted for in all multivariable analyses where needed.

The analyses were conducted separately for boys and girls following previous practices [4, 5, 8]. For each sex, interactions between race and age and race and BMI categories were also examined. Results were reported for the following age-and-sex-specific subgroups: (a) boys aged 8–12 years, (b) boys aged 13–17 years, (c) girls aged 8–12 years, and (d) girls aged 13–17 years based on the evidence of potential marginally significant interactions (P = 0.08) between race and age among boys. Significant interactions between race and BMI categories were also observed in boys aged 13–17 years and stratified analyses by BMI categories were further performed among this subgroups. Interactions between race and LBW were not explored considering the small sample size of participants born at a LBW with PHT. Analyses involving birth weight were only performed among children aged 8–15 years, the ages for which birth weight was available in NHANES.

All analyses took account of the complex survey design including stratification, clustering, and weighting to consider the oversampling of subgroups, unit non-response and non-coverage in the NHANES. This was realized by applying the set of survey commands (with prefix ‘svy-’) in STATA 12 with the sampling errors estimated by the Taylor linearization method. Statistical significance was set at the level of P ≤ 0.05 (2 tailed).

## Results

### Background Characteristics

Of 9,250 children and adolescents aged 8–17 years who were included in this analysis, 31.24% were Non-Hispanic Whites, 32.18% were Non-Hispanic Blacks, and 36.57% were Mexican Americans (Table 1). On average, Black and Mexican American participants had significantly higher mean BMI and BMI z scores compared to their White peers (mean BMI: 22.58 and 22.48, vs. 21.28, respectively, both P<0.001; BMI z score: 0.71 and 0.75, vs. 0.45, respectively, both P<0.001). Black and Mexican American youth also had significantly higher prevalence of obesity than Whites (22.88% and 24.68%, vs. 15.84%, respectively, both P<0.001). Although Blacks and Mexican Americans on average were born at significantly lower birth weight than Whites, only Blacks had a significantly higher prevalence of LBW (14.56% vs. 5.89%, P<0.001). Overall, Blacks had a higher SBP compared to Whites (107.80 vs. 106.32, P<0.001), and Mexican Americans had a lower DBP than Whites (57.90 vs. 59.25, p = 0.001). Antihypertensive medication use was low in general.

**Table 1:** Study participants' characteristics by race among children and adolescents aged 8–17 years in NHANES, 1999–2010. Estimates considering the complex survey design. SE: standard error; BMI: body mass index; SBP: systolic blood pressure; DBP: diastolic blood pressure. *P* value is for the comparison of group % or mean to that of Non-Hispanic Whites

Characteristics	Non-Hispanic Whites		Non-Hispanic Blacks			Mexican Americans		
	% or mean	SE	% or mean	SE	*P*	% or mean	SE	*P*
N (%)^†^	2,890 (31.24)	/	2,977 (32.18)	/	/	3,383 (36.57)	/	/
Age, years	12.86	0.07	12.78	0.07	0.397	12.52	0.02	0.003
Females, %	49.52	1.10	51.67	1.00	0.114	50.27	0.09	0.566
BMI, kg/m2	21.28	0.12	22.58	0.13	<0.001	22.48	0.16	<0.001
BMI z score	0.45	0.03	0.71	0.02	<0.001	0.75	0.10	<0.001
BMI >95th, %	15.84	0.93	22.88	0.82	<0.001	24.68	1.10	<0.001
Birth weight^#^, lbs	7.54	0.04	6.92	0.04	<0.001	7.36	0.04	0.001
Low birth weight^#^, %	5.89	0.59	14.56	1.06	<0.001	7.62	0.74	0.072
SBP, mm Hg	106.32	0.25	107.80	0.28	<0.001	105.95	0.30	0.294
SBP, mm Hg^£^	106.33	0.25	107.82	0.29	<0.001	105.93	0.25	0.294
DBP, mm Hg	59.25	0.32	59.50	0.38	0.570	57.90	0.34	0.001
DBP, mm Hg^£^	59.24	0.32	59.47	0.37	0.590	57.90	0.34	0.002
Antihypertensive medication use, %	0.94	0.21	0.41	0.15	0.048	0.18	0.01	0.002

^†^Non-weighted

^#^Only among children and adolescents aged 8–15 years

^£^among individuals who were not taking antihypertensive medications

### Prevalence of PHT among children and adolescents by race and other factors

In 1999–2010, the prevalence (SE) of hypertension in U.S. children and adolescents aged 8 to 17 years was 6.07% (0.39) among both sexes, 5.58% (0.49) in girls, and 6.56% (0.58) in boys. No significant difference was observed between boys and girls (P = 0.20). Mexican Americans had the lowest PHT prevalence at 5.36% (0.57), followed by 5.60% (0.55) in Non-Hispanic Whites and 7.10% (0.59) in Blacks (Table 2). Compared to non-Hispanic White youth, the unadjusted prevalence of PTH was significantly higher in non-Hispanic Blacks (P = 0.04), but the difference in Mexican Americans did not approach significance (P = 0.77). When stratified by sex alone, no racial differences were observed among boys or girls. When further stratified by age and sex, among boys aged 13–17 years Blacks had significantly higher PTH than Whites 9.59% (1.02) vs. 6.59% (1.04) (P = 0.04).

**Table 2:** Prevalence (%) of hypertension in US children and adolescents, stratified by sex and age, NHANES 1999–2010. "-" indicates no observation. *P* value is for the comparison of group % to that of Non-Hispanic Whites.

Race	N	%	SE	*P*
*All*				
All race	9,250	6.07	0.39	
Non-Hispanic Whites	2,890	5.60	0.55	
Non-Hispanic Blacks	**2,977**	**7.10**	**0.59**	**0.04**
Mexican Americans	3,383	5.36	0.57	0.77
*Boys aged 8–12 years*				
Non-Hispanic Whites	612	6.00	1.00	
Non-Hispanic Blacks	555	5.03	1.05	0.64
Mexican Americans	710	6.44	1.10	0.47
*Boys aged 13–17 years*				
Non-Hispanic Whites	831	6.59	1.04	
Non-Hispanic Blacks	**910**	**9.59**	**1.02**	**0.04**
Mexican Americans	945	6.66	0.08	0.97
*Girls aged 8–12 years*				
Non-Hispanic Whites	618	6.81	1.04	
Non-Hispanic Blacks	649	7.18	1.07	0.80
Mexican Americans	728	4.88	0.79	0.13
*Girls aged 13–17 years*				
Non-Hispanic Whites	829	4.53	0.82	
Non-Hispanic Blacks	863	6.47	0.92	0.12
Mexican Americans	1,000	3.73	0.66	0.46

### Racial Differences in PHT Prevalence and Their Association with Body Size and Birth Weight

It was further assessed whether observed racial differences in PTH can be explained by body size and birth weight using multivariate logistic regressions (Table 3). After controlling for age and sex, Black youth had a 29% increased odds of hypertension (model 1, Odds ratio (OR) = 1.29; 95% CI: 1.01–1.65; P = 0.044) compared to White youth. The Black-White difference in hypertension rate showed little change (model 2, OR = 1.25; P = 0.047) after additional adjustment for birth weight, but was attenuated (model 3, OR = 1.16; P = 0.19) to the degree that it lost statistical significance with further adjustment for current BMI. When stratified by sex and age group combined, no significant racial differences were identified in age adjusted models and after controlling for BMI categories, LBW status and their interaction among boys aged 8–12 years. However, among boys aged 13–17 years Blacks were at significantly higher odds of PHT compared to Whites in the age adjusted model (OR: 1.51; 95% CI: 1.03–3.43; P = 0.041) and with the addition of control for LBW (OR: 2.00; 95% CI: 1.09–3.71; P = 0.027), and then for BMI categories (OR: 1.50; 95% CI: 1.01–2.14; P = 0.048). There was no significant racial difference among girls for both 8–12 and 13–17 age groups. No significant differences in PTH were observed between White and Mexican youth in any models including those for each sex-age group.

**Table 3:** Odd ratio for hypertension in Non-Hispanic Blacks and Mexican Americans compared to Non-Hispanic Whites among children and adolescents in NHANES, 1999–2010. Model 1: controlled for age and sex. Model 2: model 1 + low birth weight. Model 3: model 1 + current BMI categories

Characteristics	Model 1		Model 2		Model 3	
	OR (95% CI)	*P*	OR (95% CI)	*P*	OR (95% CI)	*P*
*All participants*						
Non-Hispanic Whites	1	/	1	/	1	/
Non-Hispanic Blacks	**1.29 (1.01–1.65)**	**0.04**	**1.25 (1.01–1.60)**	**0.04**	1.16 (0.93–1.45)	0.19
Mexican Americans	0.95 (0.70–1.29)	0.72	0.94 (0.69–1.27)	0.70	0.82 (0.63–1.08)	0.16
*Boys aged 8–12 years*						
Non-Hispanic Whites	1	/	1	/	1	/
Non-Hispanic Blacks	0.88 (0.52–1.49)	0.65	0.82 (0.48–1.41)	0.47	0.85 (0.50–1.44)	0.55
Mexican Americans	1.09 (0.66–1.78)	0.74	1.05 (0.64–1.72)	0.84	0.97 (0.60–1.56)	0.90
*Boys aged 13–17 years*						
Non-Hispanic Whites	1	/	1	/	1	/
Non-Hispanic Blacks	**1.51 (1.03–3.43)**	**0.04**	**2.00 (1.09–3.71)**	**0.02**	**1.50 (1.01–2.14)**	**0.04**
Mexican Americans	1.00 (0.63–1.58)	0.99	1.31 (0.68–2.51)	0.42	0.87 (0.54–1.40)	0.56
*Girls aged 8–12 years*						
Non-Hispanic Whites	1	/	1	/	1	/
Non-Hispanic Blacks	1.05 (0.68–1.65)	0.81	0.98 (0.62–1.566)	0.96	0.98 (0.62–1.55)	0.92
Mexican Americans	0.70 (0.44–1.10)	0.12	0.66 (0.42–1.04)	0.07	0.66 (0.41–1.06)	0.09
*Girls aged 13–17 years*						
Non-Hispanic Whites	1	/	1	/	1	/
Non-Hispanic Blacks	1.48 (0.92–2.37)	0.81	1.75 (0.95–2.99)	0.07	1.33 (0.82–2.17)	0.24
Mexican Americans	0.83 (0.48–1.43)	0.50	0.97 (0.45–2.09)	0.94	0.79 (0.46–1.35)	0.39

**Fig 1:**
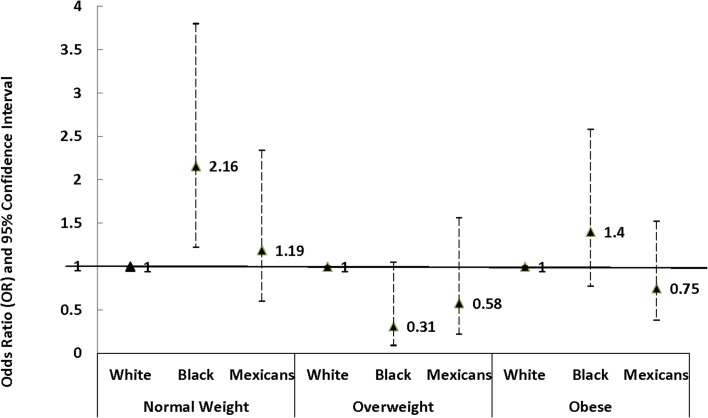
Odd ratio of hypertension in Non-Hispanic Blacks and Mexican Americans compared to Non-Hispanic White among boys aged 13–17 years in NHANES, 1999–2010, stratified by BMI category.

Further analysis stratified by the BMI categories among boys aged 13–17 years shows that significant Black-White differences were only found in boys with normal body size (OR = 2.16; 95% CI: 1.22–3.80; P = 0.008), but not among those who were overweight or obese (Fig 1). No significant differences in PTH were observed between Whites and Mexican across all BMI categories. When the overweight and obese categories were combined to potentially increase statistical power, the results remain the same (OR: 1.03; 95% CI: 0.65–1.66; P = 0.91 in Black and OR: 0.71; 95% CI: 0.44–1.13; P = 0.15 in Mexicans).

## Discussion

The study investigated racial differences in the prevalence of PTH among children and adolescents aged 8–17 years and examined their association with body size and birth weight using the nationally representative sample of NHANES 1999–2010. The prevalence of hypertension in U.S. children and adolescents aged 8 to 17 years was 6.1%. We found that Black youths had a 29% increased odds of hypertension compared to their White peers. When stratified by sex and age groups, the Black-White difference was only significant in boys aged 13–17. Compared to Whites, Black youths also had an elevated prevalence of obesity and lower birth weight. However, the racial difference in PTH among boys aged 13–17 was not explained by their obesity and low birth weight status. No significant differences in PTH were observed between White and Mexican s youth taken as a whole or by sex-age group.

It is well documented that in the US Black adults have higher prevalence of hypertension than White adults. However, it is unclear whether such racial disparity also exists in US children and adolescents. It is also unknown at which age the Black-White differences in BP emerge. While most previous studies found no significant racial differences in hypertension among US youths, Rosner and colleagues reported a significant racial difference between Hispanic and White boys, but not between Black and White boys [5]. For girls, both Blacks and Hispanics were found to have higher prevalence as compared to their White peers. In contrast, we found a Black-White difference in PTH among US youths in the current study. When stratified by age and sex, such a racial difference was only significant among boys aged 13–17, which persisted after controlling for birth weight and BMI. Although Mexican American youth had a higher rate of LBW and obesity compared to Whites, they had little difference in PTH after adjusting for age, sex, birth weight and obesity. The differences in BP measurement and hypertension definition might partially explain the differences in findings between the current and Rosner’s study. The timing of when the study data were collected may also influence the two studies' results. The Rosner study dataset encompassed all children in the Pediatric Task Force Database which included surveys and studies conducted between 1973 and 2000. Our study, however, applied the most recent national representative sample collected between 1999 and 2010. Our results are consistent with 3 recent studies in US populations [9, 10, 17] and provided additional data stratified by age and gender. Nevertheless, more studies are warranted to shed further light on the racial differences in pediatric hypertension prevalence in the US.

Another important finding of this study is the Black-White disparity was observed among adolescent boys with normal weight, but not among overweight and obese boys. Similar findings that Blacks at normal weight were more likely to have PHT than Whites have been reported previously [4, 5]. However, the racial difference was significant among female adolescents in Rosner et al. [4], and male children and adolescents in Rosner et al. [5]. It is unclear why among Black adolescents only those at normal weight, but not those overweight or obese were at increased risk of PTH than their White peers. Although we cannot rule out the possibility that the lack of significant results in overweight and obese groups is due to smaller sample size, results were similar when we combined the overweight and obese youths together.

The current study has several limitations. First, the 3 BP measurements were all obtained in one visit during physical examinations in the NHANES. Ideally, hypertension should be defined according to 3 repeated BP measures obtained on 3 separate occasions [19]. Secondly, the reliability of our findings in analyses stratified by age-sex group could be compromised by small sample size, although each age-sex group consisted of at least 600 individuals. Thirdly, NHANES is a cross-sectional survey by design, which is not the best design to examine the age onset of racial disparity in PTH. Lastly, data on LBW were self-reported and only available among participants aged 8–15 years. Misclassification in self-reported birth weight, although likely small, could introduce bias into the association between LBW and PHT. Further, when controlling for LBW the study sample was limited to those aged 8–15 years. A sensitivity analysis demonstrated that restricting all analyses to that age range yield qualitative unchanged results. Future studies are warranted to control for a better measured LBW status.

Despite these limitations, this study contributes to the literature by examining the most recent nationally representative samples of the US population including extensive relevant data with PTH defined using three measurements of BP. Other notable advantages of this study include the relatively large sample size available in each race group, objective measure of weight and height, and the combination of stratified and multivariate analyses guided by both previous studies and empirical data (test for interactions).

## Conclusions

In current analysis of the most recent nationally representative samples of US children and adolescents aged 8–17 years, we found Non-Hispanic Black children and adolescents to have a significantly higher prevalence of hypertension than their non-Hispanic White counterparts. This racial difference in pediatric hypertension was only shown in boys aged 13–17 when stratified by age and sex. The racial disparity of PTH among boys aged 13–17 could not be explained by low birth weight and current obesity of the Black youth. No significant differences in PTH were observed between White and Mexican Americans in all models and in each age-sex group. Our findings are not entirely consistent with previous findings. Future research is needed to further investigate racial differences in hypertension in children and adolescents.
